# Comparison of multi-track mini single arm external fixation and plate internal fixation in the treatment of missed Monteggia fracture in children

**DOI:** 10.3389/fped.2026.1795705

**Published:** 2026-06-19

**Authors:** Rui Bai, Xiaoli Ding, Hongbo Chen, Zhenyu Bai, Long Wu, Peng Ye

**Affiliations:** Department of Orthopedics, General Hospital of Ningxia Medical University, Yinchuan, China

**Keywords:** children, external fixation, fracture fixation, missed Monteggia fracture, ulnar osteotomy

## Abstract

**Objective:**

To analyze the effect of multi-track mini single arm external fixation and plate internal fixation in the treatment of missed Monteggia fracture in children.

**Methods:**

Retrospectively analyzed the data of 22 children with missed Monteggia fracture admitted to the General Hospital of Ningxia Medical University from April 2018 to October 2024. There were 14 males, 8 females, 5 on the left side, and 17 on the right side. Age (5.3 ± 1. 9) years old at the time of injury, 17 cases of Radiocapitellar dislocation forward, 1 case of posterior dislocation, and 4 cases of anterolateral dislocation. The time from injury to surgery was (31.0 ± 11.5) months, and all patients were treated with Boyd's approach multi-track mini single arm external fixation or plate internal fixation. The range of elbow flexion and extension, the range of forearm rotation, the length of operation and the volume during operation, the range of elbow flexion and extension, the range of forearm rotation, the Mayo score of elbow and the healing time of osteotomy were measured and compared in all patients. At the same time, the incidence of complications was recorded.

**Results:**

All patients were followed up for 12 months. There was no statistical difference between the two groups in the range of motion of the elbow joint before operation (*p* > 0.05). The duration of operation (97.7 ± 4. 9 min) and the volume during operation (47.4 ± 6. 4 mL) in the external fixation group were smaller than those in the internal fixation group (132.8 ± 8. 5 min, 70.3 ± 16. 1 mL), the difference was statistically significant (*p* < 0.05); At 12 months after operation, the elbow flexion range (127.8 ± 5. 9°), forearm pronation (81.2 ± 3. 3°), and forearm supination (84.2 ± 3. 2°) in the external fixation group were significantly improved compared with the internal fixation group (121.6 ± 7. 1°, 73.2 ± 5. 1°, 80.1 ± 5. 4°), the difference was statistically significant (*p* < 0.05), and the elbow extension range (0.7 ± 7. 8°) in the external fixation group was not significantly different from the internal fixation group (5.9 ± 8. 3°) (*p* > 0.05); The Mayo score of elbow joint function in the external fixation group (92.0 ± 7. 1) was better than that in the internal fixation group (86.7 ± 9. 8), the difference was statistically significant (*p* < 0.05); There was no significant difference in osteotomy healing time between external fixation group (6.8 ± 1. 0 months) and internal fixation group (5.8 ± 1. 6 months) (*p* > 0.05); The overall complication rate of the two groups was significantly higher in the external fixation group (3 (30.0%)) than that in the internal fixation group (5 (41.7%)) (*p* > 0.05).

**Conclusion:**

Both multi-track mini single arm external fixation and plate internal fixation can effectively treat the missed diagnosis of missed Monteggia fracture in children. The former may have more prominent advantages in operative efficiency and postoperative recovery of elbow flexion and forearm rotation.

## 1 Introduction

Monteggia Fracture is a complex elbow joint injury involving proximal ulna fractures with Radiocapitellar dislocation, which is particularly common in children and adolescents (1). If the early diagnosis and proper treatment are not timely, the injury will progress to missed Monteggia fracture after more than 4 week (2–4). The main pathological changes include malunion of the ulna (such as angulation or shortening), Radiocapitellar dislocation, and then lead to dysfunction and deformity of the elbow joint (5). Although there are many studies on the treatment strategies of missed Monteggia fracture (such as osteotomy, fixation methods and annular ligament management), which option is the most optimal is still inconclusive, especially in the choice of fixation (6). To this end, the purpose of this study was to systematically compare the clinical results of missed Monteggia fracture treated with plate internal fixation and multi-track mini single-arm external fixation after ulnar osteotomy.

## 2 Information and methodology

### 2.1 Case inclusion and exclusion criteria case inclusion criteria

① Have a clear history of trauma and the injury time is ≥ 4 weeks; ② Patients with Radiocapitellar dislocation combined with ulnar deformity; ③ Age ≤ 14 years old at the time of initial operation; ④ Using Boyd's approach multi-track mini single arm external fixation or plate internal fixation; There are detailed and true medical history data in the medical record system. Exclusion criteria: ① Patients with a history of surgery in the affected limb, which affected the prognosis of missed Monteggia fracture in children; ② Congenital Radiocapitellar dislocation or congenital forearm deformity and dysfunction; ③ Brachio-ulnar joint and superior ulnar-radial joint are severely deformed; ④ Patients with ulnar and radial infection, tumors and other diseases; ⑤ Incomplete follow-up data.

### 2.2 General information

This study is a preliminary retrospective pilot study, including 22 patients, 14 males and 8 females; Age at the time of injury (5.3 ± 1. 9) years old, 17 cases of anterior Radiocapitellar dislocation, 1 case of posterior dislocation, 4 cases of anterolateral dislocation; The time from injury to surgery was (31.0 ± 11.5) months. All patients were fall injury, no combined injury, preoperative complete bilateral elbow joint anteroposterior and lateral and forearm full-length x-ray film.

### 2.3 Surgical methods

#### 2.3.1 External fixation group

The child was taken into supine position, and after satisfactory general anesthesia, a sterile tourniquet was ligated on the proximal end of the upper arm. An oblique incision was made from the lateral epicondyle of the humerus to the olecranon of the ulna through the natural space between the extensor carpi ulnaris and the elbow muscles, and the posterior joint capsule was incised to fully expose the proximal end of the ulna, Radiocapitellar dislocation. Firstly, the proximal ulnar osteotomy was performed according to the preoperative plan, and three and a half nails were placed at the far and near ends of the osteotomy line, and a multi-track mini single-arm external fixation frame was installed. Explore the internal structure of the Radiocapitellar joint, repair or reconstruct the scarred annular ligament, and remove the fibrous scar tissue that hinders the reduction of the Radiocapitellar joint. Retraction of the radial head under direct vision can be tried out, and if necessary, the anatomical reduction of the radial head can be realized by using instruments to pry. Then, the external fixator is adjusted to lengthen the osteotomy end and angle it moderately backward, thereby restoring the physiological length and curvature of the ulna. The elbow joint was mobilized to assess the stability of the Radiocapitellar joint and to evaluate the fixation firmness of the osteotomized end. If the stability is not good, the proximal ulna is fixed with an elastic intramedullary nail, which passes through the osteotomy to enhance the overall stability. According to the joint of the osteotomy end and the degree of bone defect, whether to do autologous iliac bone grafting or artificial bone grafting was decided. Postoperative elbow flexion 90° forearm supination long arm plaster fixation. The osteotomy end was gradually lengthened by adjusting the external fixator 1 week after operation until the anatomical length of the ulna was restored. At 6 weeks after operation, the elbow joint function exercises were performed gradually.

#### 2.3.2 Internal fixation group

After general anesthesia, Boyd's approach was used. After incision of the skin, subcutaneous and deep fascia, the joint capsule was cut through the space between the extensor carpi ulnaris and the elbow muscle, and the proximal ulna, the Radiocapitellar joint and the Radiocapitellar dislocation were exposed. After proximal ulnar osteotomy (7, 8), the scarred annular ligament was repaired or reconstructed, the scar tissue of the joint was removed, and the radial head was reduced under direct vision. After confirming the stability of the Radiocapitellar joint, the length and physiological radian of the ulna were restored by lengthening and angling the ulnar osteotomy end, and then internal fixation with the plate and screw system. During the operation, x-ray films of the elbow joint including the forearm were taken to confirm that the Radiocapitellar joint was well aligned. After operation, the elbow was flexed 90° forearm supination plaster fixation. After 6 weeks, the elbow joint function exercise was carried out gradually.

#### 2.3.3 Postoperative management

Antibiotics were given to prevent infection after operation, regular review was performed to monitor the alignment of the Radiocapitellar joint and bone healing, and to evaluate the removal of internal or external fixation devices after ulna healing.

### 2.4 Curative effect evaluation

#### 2.4.1 Record the duration of operation and intraoperative volume of all patients

The range of motion of elbow joint was measured by hand-held measuring instrument before and 12 months after operation, including flexion, extension, pronation and supination angles; The incidence of postoperative complications, including brachioradial joint dislocation, incision/needle tract infection, delayed union or non-union, nerve injury, whether Radiocapitellar joint fixation; Osteotomy healing time; And make a comparative analysis.

#### 2.4.2 Measure the elbow

Mayo score 12 months after operation, the scale includes 4 dimensions: ① pain: 0–45 points, the lower the score, the more severe the pain; ② Motion range: 0–20 points, the lower the score, the smaller the curvature range of elbow joint; ③ Stability: 0–10 points, the lower the score, the greater the degree of activity restriction; ④ Daily function: 0∼25 points, the lower the score, the greater the degree of life restriction of children. The full score is 100 points, ≥ 90 is excellent, 75 ∼ < 90 is good, 60 ∼ < 75 is average, and < 60 is poor (9–11).

### 2.5 Statistical methods

The data were calculated and analyzed with SPSS 27.0 software, and the measurement data were expressed by t-test and mean ± standard deviation (x ± s); The count data were expressed by *χ*2 test and rate (%), *P* < 0.05 indicated that the difference was statistically significant.

## 3 Result

### 3.1 Comparison of the general data of the two groups of patients

There was no significant difference between the two groups (*p* > 0.05) in terms of sex, side of affected limb, age at the time of injury and time from injury to operation (Table 1).

**Table 1:** Comparison of general information.

Category	Mini external fixation group (*n* = 10)	Plate internal fixation group (*n* = 12)	Statistic	*p* value
Gender (M/F)	5/5	9/3	–	0.390
Affected limb (L/R)	3/7	2/10	–	0.633
Age at time of injury (Months)	60.1 ± 20.3	66.3 ± 24.1	t = −0.553	0.532
Age at time of injury (Months)	30.9 ± 8.7	31.0 ± 12.4	U = 61.500	0.984

### 3.2 Comparison of operation duration, intraoperative volume, complications and healing between the two groups

The duration of operation (97.7 ± 4.9 min) and intraoperative volume (47.4 ± 6.4 mL) in the external fixation group were significantly lower than those in the internal fixation group (132.8 ± 8.5 min, 70.3 ± 16.1 mL) (*P* < 0.05); The overall complication rate of the external fixation group (3 (30.0%)) was better than that of the internal fixation group (5 (41.7%)), but the difference was not statistically significant (*P* > 0.05); There was no significant difference in osteotomy healing time between the external fixation group (6.8 ± 1. 0 months) and the internal fixation group (5.8 ± 1. 6 months) (*P* > 0.05) (Table 2).

**Table 2:** Comparison of operation duration, intraoperative volume, complications and healing.

Category	Mini external fixation group (*n* = 10)	Plate internal fixation group (*n* = 12)	Statistic	*p* value
Length of operation (min)	97.7 ± 4.9	132.8 ± 8.5	t = −11.764	<0.001
Intraoperative volume (ml)	47.4 ± 6.4	70.3 ± 16.1	U = 5.000	<0.001
Overall complications [*n* (%)]	3 (30.0%)	5 (41.7%)		0.673
Radiocapitellar redislocation^*^	1/10 (10.0%)	3/12 (25.0%)		
Delayed/nonunion of ulna	1/10 (10.0%)	2/12 (16.7%)		
Radiocapitellar joint fixation	1/10 (10.0%)	0/12 (0%)		
Nerve injury	1/10 (10.0%)	1/12 (8.3%)		
Osteotomy healing time (months)	6.8 ± 1.0		t = 1.885	0.092

* Some patients have multiple complications.

### 3.3 Comparison of curative effects between the two groups

There was no significant difference between the two groups in elbow flexion and extension and forearm rotational mobility before operation (*P* > 0.05); The elbow flexion range (127.8 ± 5. 9°), forearm pronation (81.2 ± 3. 3°), and forearm supination (84.2 ± 3. 2°) in the external fixation group were significantly improved compared with the internal fixation group (121.6 ± 7. 1°, 73.2 ± 5. 1°, 80.1 ± 5. 4°), the difference was statistically significant (*p* < 0.05), and the elbow extension range (0.7 ± 7. 8°) in the external fixation group was not significantly different from the internal fixation group (5.9 ± 8. 3°) (*p* > 0.05); The Mayo score of elbow joint function in the external fixation group (92.0 ± 7. 1) was better than that in the internal fixation group (86.7 ± 9. 8), the difference was statistically significant (*P* < 0.05) (Table 3).

**Table 3:** Comparison of elbow and forearm mobility and elbow Mayo function score before and after operation.

Category	Mini external fixation group (*n* = 10)	Plate internal fixation group (*n* = 12)	Statistic	*p* value
Preoperative mo				
Flexion	91.8 ± 10.3	89.0 ± 7.1	t = 0.740	0.468
Straightening	6.4 ± 16.3	8.9 ± 12.6	t = −0.402	0.689
Pronation	69.1 ± 7.9	63.4 ± 6.6	t = 0.777	0.067
Supination	77.5 ± 3.5	77.3 ± 6.2	t = 0.106	0.931
Postoperative mobility(°)				
Flexing	127.8 ± 5.9	121.6 ± 7.1	t = 4.806	0.028
Straightening	0.7 ± 7.8	5.9 ± 8.3	t = −1.609	0.132
Pronation	81.2 ± 3.3	73.2 ± 5.1	U = 108.500	<0.001
Supination	84.2 ± 3.2	80.1 ± 5.4	t = 2.269	0.038
Mayo total score	92.0 ± 7.1	86.7 ± 9.8	U = 101.000	0.045

### 3.4 Typical cases

**Figure 1:**
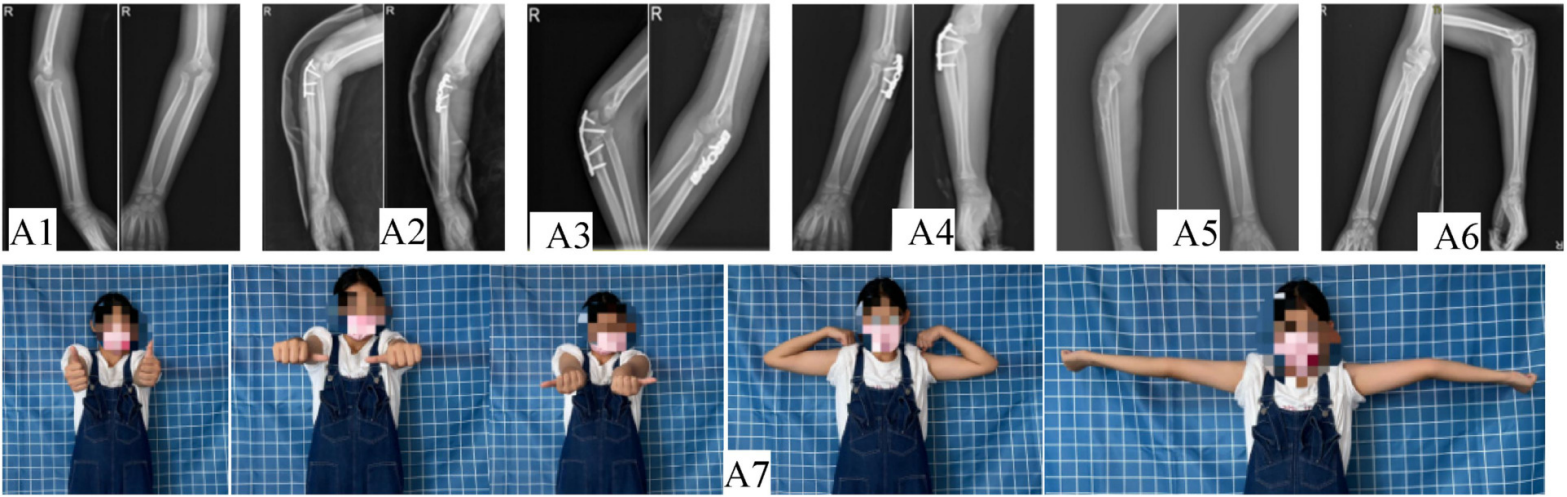
Follow-up photos of plate fixation.

① A 7-year-old woman with an missed Monteggia fracture was diagnosed and treated in time. Three months after the injury, she underwent ulnar osteotomy via Boyd's approach, plate internal fixation, annular ligament exploration, and plaster fixation. Anteroposterior x-ray of elbow joint before operation (Figure 1A1). Radiocapitellar dislocation, postoperative anteroposterior x-ray of elbow joint (Figure 1A2). Radiocapitellar joint alignment was good, postoperative reexamination 1 month (Figure 1A3). Ulnar osteotomy fixation was reliable and Radiocapitellar joint alignment was good, postoperative reexamination 6 months (Figure 1A4). After osteotomy healing was good, plate removal reexamination (Figure 1A5). Radiocapitellar joint alignment was good. After 12 months (Figure 1A6), the recovery was good, and the range of motion of elbow flexion and extension and forearm rotation (Figure 1A7) was measured with a soft ruler, and the range of motion was not significantly limited.

**Figure 2:**
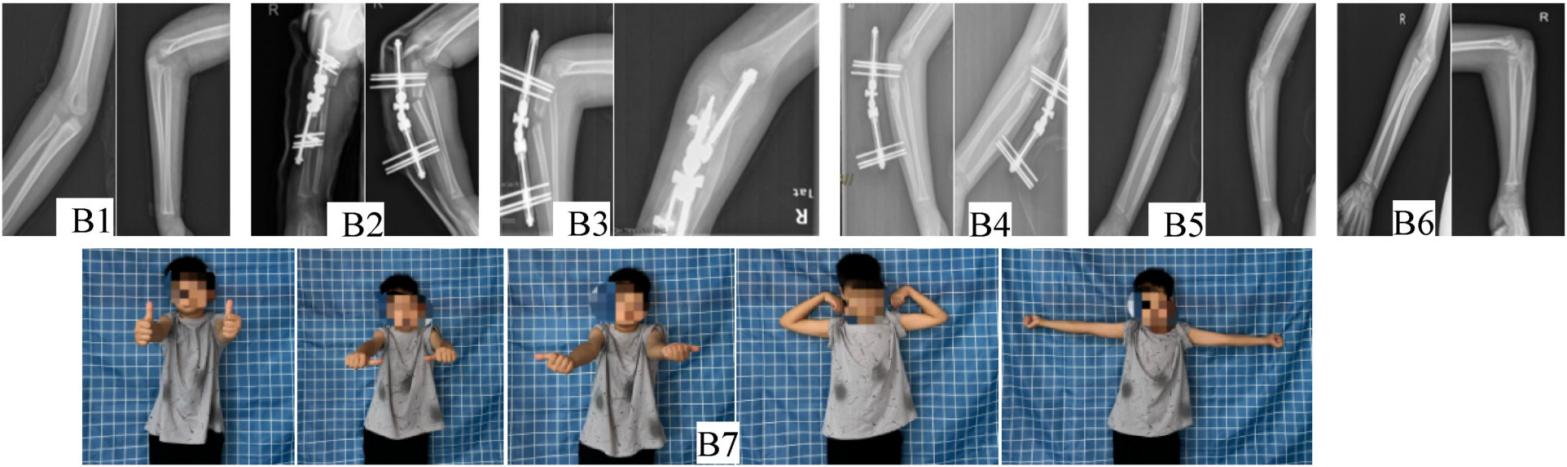
Follow-up photo of multi-track mini single-arm external fixation treatment.

② Timely diagnosis and treatment of a 3-year-old male with missed Monteggia fracture. Three months after injury, the patients were treated with ulnar osteotomy by Boyd's approach, ring ligament exploration, multi-track mini single arm external fixation and plaster fixation. Anteroposterior x-ray film of the elbow joint before operation (Figure 2B1). Radiocapitellar dislocation, anteroposterior x-ray film of the elbow joint on the second day after operation (Figure 2B2). Radiocapitellar joint alignment was good, reexamination 1 month after operation (Figure 2B3). Ulnar osteotomy fixation was reliable Radiocapitellar joint alignment was good, reexamination 7 months after operation (Figure 2B4). Osteotomy healing was good, reexamination after taking out the multi-track mini single arm external fixator (Figure 2B5). Radiocapitellar joint alignment was good. After 12 months (Figure 2B6), the recovery was good, and the range of motion of elbow flexion and extension and forearm rotation (Figure 2B7) was not significantly limited.

## 4 Discussion

The main pathological changes of missed Monteggia fracture are Radiocapitellar dislocation and its secondary changes and ulnar angulation. At this time, the Radiocapitellar joint cannot be satisfactorily reduced by manipulation, manifesting as sexual dislocation of the radial head, which leads to unbalanced growth of the ulna and radius. After the dislocation of the radial head, the normal alignment relationship of the Radiocapitellar joint is lost, and the shape of the radial head changes due to the lack of compressive stress of the humeral head, gradually changing from the original round concave shape to an abnormal shape that is flat or even protruding. At the same time, malunion of the ulna occurs, which eventually leads to forearm deformity, elbow flexion, and limited forearm rotation (12–14). In addition, ulnar malunion often manifests as abnormal arcuation, and this structural change is one of the important factors hindering the reduction of the radial head (15–17).

### 4.1 Management of annular ligament

At present, the treatment strategy of annular ligament in the surgical treatment of missed Monteggia fracture has not yet reached a unified consensus in clinical practice. Some scholars believe that intraoperative repair or reconstruction of the annular ligament can enhance the stability of the radial head after surgery (18). At present, a variety of reconstruction techniques have been applied in clinical practice, such as triceps brachii tendon transposition, fascia strip reconstruction, and free palmaris longus tendon graft (19–21). In this study, initially we tried to perform a closed reduction of the dislocated radial head, but successful reduction was difficult. For patients with difficult closed reduction, we take open reduction of the radial head. In patients with mild torn annular ligament and structural integrity, we give priority to trying to repair the annular ligament in order to restore its physiological stability. However, due to the prolonged course of old injuries, severe scarring often occurs in annular ligaments, and it is even difficult to identify (22). In order to achieve the reduction of the radial head, it is sometimes necessary to remove the fibrous scar tissue that hinders the reduction, and the highly scarred annular ligament is often difficult to completely peel off, or is misjudged as an obstructive structure during the operation and removed together, making primary ligament repair in missed Monteggia fracture face greater challenges (8, 23). Some studies have pointed out that the stability of the radial head cannot be completely guaranteed by repairing or reconstructing the annular ligament alone, and the stability of the radial head after reduction can be further enhanced by restoring the tension of the interosseous membrane through osteotomy and orthopedics of the deformed ulna (5, 24–26). In addition, annular ligament reconstruction often results in restricted forearm rotation (especially pronation), and therefore, annular ligament reconstruction is not necessarily (13, 27, 28). Comprehensive consideration, in the treatment of missed Monteggia fracture in children, whether to reconstruct the annular ligament should be individualized according to the specific conditions during the operation (12).

### 4.2 Selection of ulnar osteotomy and fixation

At present, the treatment of missed Monteggia fracture using multi-track mini single arm external fixation or plate internal fixation has been reported in clinical practice (27, 29). Internal fixation with plate after ulnar osteotomy can provide strong internal fixation for the broken end of ulnar osteotomy and is convenient for postoperative management, but it has high requirements for the operator and surgical technique. In addition, if there is a slight displacement of the broken end after intraoperative plate fixation, it is usually difficult to go further, and a second operation is required to remove the internal fixation after the fracture healing. Some scholars use multi-track mini single-arm external fixation to treat such fractures. According to the preoperative plan, half nails are placed at the far and proximal ends of the osteotomy area, and the remaining half nails are placed after the external fixator is installed. The shape of the external fixator matches the shape of the ulna., and then complete the osteotomy at the predetermined position. Afterwards, the angulation and lengthening correction of the ulna were achieved by external fixation. The advantage of this method is that the osteotomy fracture can still be accurately aligned by the stent in the coronal and sagittal positions after fixation. However, the fixation strength of the external fixator is usually weaker than that of the plate, so it can be assisted by Kirschner wires to enhance the fixation effect in cases where the broken end is judged to be extremely unstable during the operation.

### 4.3 Analysis of the results of this study

The results of this study indicate that the treatment of missed Monteggia fracture in children with multi-track mini single-arm external fixation, compared with plate internal fixation, shows significant advantages in terms of shortening surgical time, intraoperative and complication rates, as well as improving postoperative joint function. In the external fixation group, the surgical incision was smaller, and there was no need for extensive dissection of soft tissue and periosteum, and the local blood supply was smaller, which directly led to a significant shortening of intraoperative volume and operative time, and at the same time created more favorable results for fracture healing and functional recovery. The better functional outcome of the elbow joint in the external fixation group may be due to the following aspects: ① The elastic fixation provided by the external fixator makes the osteotomy fracture end produce beneficial fretting, which not only promotes local blood supply and bone healing, but also allows patients to perform early postoperative functional exercises, which effectively reduces the risk of joint stiffness. ② The external fixator has the unique advantage of being adjustable during and after operation. Intraoperatively, it allows precise alignment of the osteotomy end in both sagittal and coronal positions to reconstruct the ulnar arch, thereby enhancing the stability of the Radiocapitellar joint by restoring interosseous membrane tension (30). If the radial head is not reduced during the operation, the ulna can still be gradually lengthened by the external fixator after the operation to achieve concentric reduction of the Radiocapitellar joint (31). ③ Gradually lengthening the osteotomy end after surgery can overcome soft tissue contracture, the risk of delayed union or non-union, and the incidence of nerve palsy and compartment syndrome (31). In contrast, as a static internal fixation, the plate cannot be performed once it is placed. In the aspect of osteotomy healing, the time of external fixation group was slightly longer than that of internal fixation group, but there was no significant difference between the two groups as a whole. In terms of complications, the overall incidence of the external fixation group, especially the Radiocapitellar dislocation rate, was significantly lower than that of the internal fixation group. In this study, one patient with Radiocapitellar joint re-dislocation occurred in the external and external fixation group. After enlarging the posterior-lateral angle of the ulna, stable reduction was achieved. Sometimes the radial head cannot be reduced to restore the normal anatomical shape of the ulna, and the ulna deformity needs to be corrected to restore the reduction space of the radial head and reduce the radial head (32). In 1 case, the stability of osteotomy site was poor after intraoperative reduction, and Kirschner wire was used to assist the fixation and stabilize the fixation. In the internal fixation group, 3 patients had redislocation of the Radiocapitellar joint during the operation, 1 patient was reduced after rebuilding the tension of the annular ligament, and 2 patients were successfully reduced after reclearing the gap between the Radiocapitellar joint. In addition, we have observed clinically that patients with a long time from injury to surgery often have severe secondary pathological changes in the Radiocapitellar joint and the superior radioulnar joint (33). Some patients are difficult to achieve stable reduction due to the deformation of the humeral head or radial head morphology. Although the shape of the radial notch is repaired as much as possible during the operation to restore the anatomical alignment, the radial notch often has hyperosteogeny and morphological abnormalities due to dislocation, and further repair is required. To rebuild joint matching, this significantly increases the difficulty of surgery (34). Compared with the plate internal fixation, the removal of the external fixator can be completed in the outpatient clinic, which is a financial burden for the patient's family. However, external fixation may cause psychological rejection in some pediatric patients, which is a potential shortcoming of this technique.

### 4.4 Limitations of this study

The main limitations of this study are small sample size and short follow-up time. Although there were no significant differences in the basic data between the two groups, a larger sample size helped to verify the universality of the conclusions. Secondly, the success of external fixation techniques depends to a certain extent on the operator's operation and individual differences, which may have an impact on the results. In conclusion, in the ulnar osteotomy and fixation of missed Monteggia fracture, both fixations have therapeutic effects, but multi-track mini-single-arm external fixation may be superior to plate internal fixation in terms of surgical efficiency, postoperative elbow flexion and forearm rotational mobility, and complication control.

## Data Availability

The raw data supporting the conclusions of this article will be made available by the authors, without undue reservation.

## References

[1] Fan Y Liu Q Yu X Zhang J Wang W Liu C. Ultrasound, a new adjuvant method for treating acute Monteggia fracture in children. J Orthop Surg Res. (2023) 18(1):595. 10.1186/s13018-023-04075-y 37568239 PMC10422793

[2] Wang W Xiong Z Huang D Li Y Huang Y Guo Y. Risk factors for unsuccessful reduction of chronic Monteggia fractures in children treated surgically. Bone Jt Open. (2024) 5(7):581–91. 10.1302/2633-1462.57.BJO-2024-0004.R2 38991554 PMC11247538

[3] Gopinathan NR Rangasamy K Vatsya P Behera P. Management of missed type-2 Monteggia fracture equivalent in a 9-year-old child: a case report. JBJS Case Connect. (2021) 11(1):e20.00179. 10.2106/JBJS.CC.20.00179 33577186

[4] Shravan YC Prakash S Mahesh M Salutagi P. Sculpting solutions: 3D-printed models transform osteotomy planning in Monteggia fractures. J Orthop Case Rep. (2025) 15(6):162–7. 10.13107/jocr.2025.v15.i06.5702 40520726 PMC12159626

[5] Li J Zhao X Rai S Ding Y Zhang Q Ze R. Two-stage strategy for neglected Monteggia fracture in children: a retrospective study of 51 patients. Medicine (Baltimore). (2021) 100(10):e25129. 10.1097/MD.0000000000025129 33725914 PMC7969315

[6] Alajmi TAS. Neglected Monteggia fracture dislocations in children: a case series. J Orthop Case Rep. (2020) 10(7):57–62. 10.13107/jocr.2020.v10.i07.1918 33585318 PMC7857649

[7] Zhou W Li L Mu M. Treatment of missed Monteggia fracture with intact annular ligament after an interval of 9 years: a case report and literature review. J Int Med Res. (2020) 48(8):300060520949079. 10.1177/0300060520949079 32814487 PMC7444132

[8] Gryson T Van Tongel A Plasschaert F. The management of chronic paediatric Monteggia fracture-dislocation. J Orthop. (2021) 24:65–76. 10.1016/j.jor.2021.02.009 33679030 PMC7906882

[9] Ma C-H Hsueh Y-H Wu C-H Yen C-Y Tu Y-K. Does an internal joint stabilizer and standardized protocol prevent recurrent instability in complex persistent elbow instability? Clin Orthop Relat Res. (2022) 480(7):1354–70. 10.1097/CORR.0000000000002159 35266916 PMC9191335

[10] Loose O Morrison SG Langendoerfer M Eberhardt O Wirth T Fernandez FF. Radial head distalisation with an external ring fixator as a therapy option in children with chronic posttraumatic radiocapitellar dislocations. Eur J Trauma Emerg Surg. (2023) 49(4):1803–10. 10.1007/s00068-022-02173-w 36422659

[11] He JP Hao Y Shao JF. Comparison of treatment methods for pediatric Monteggia fracture: met vs missed radial head dislocation. Medicine (Baltimore). (2019) 98(2):e13942. 10.1097/MD.0000000000013942 30633171 PMC6336613

[12] Yi Y Liu C Xu Z Xie Y Cao S Wen J. What do we need to address when we treat neglected Monteggia fracture in children. Front Pediatr. (2024) 12:1430549. 10.3389/fped.2024.1430549 39268364 PMC11390576

[13] Goyal T Arora SS Banerjee S Kandwal P. Neglected Monteggia fracture dislocations in children: a systematic review. J Pediatr Orthop B. (2015) 24(3):191–9. 10.1097/BPB.0000000000000147 25714935

[14] Nishimura M Itsubo T Horii E Hayashi M Uchiyama S Kato H. Tardy ulnar nerve palsy caused by chronic radial head dislocation after Monteggia fracture: a report of two cases. J Pediatr Orthop B. (2016) 25(5):450–3. 10.1097/BPB.0000000000000302 26986030

[15] Liu QL Zhang YB Shi LY Wang Q Geng PS Wang PL. Meta analysis of surgical treatment for old Monteggia fracture in children. Zhonghua Yi Xue Za Zhi. (2018) 98(38):3096–101. 10.3760/cma.j.issn.0376-2491.2018.38.011 30392271

[16] Matheron G Wadia F Eastwood D Spanoudakis E Chui K Segaren N. Monteggia fracture-dislocations in children: a structured approach to management. Eur J Orthop Surg Traumatol. (2025) 36(1):24. 10.1007/s00590-025-04597-7 41307724

[17] Xu P Zhang Z Ning B Wang D. Outcomes and experience after open reduction for chronic Monteggia fracture in children. Transl Pediatr. (2022) 11(7):1122–9. 10.21037/tp-21-614 35958014 PMC9360808

[18] Mohan Kumar EG Yathisha Kumar GM Noorudheen M. Functional outcome of bell Tawse procedure for the management of chronic unreduced Monteggia fracture-dislocation in children. Indian J Orthop. (2019) 53(6):745–50. 10.4103/ortho.IJOrtho_47_19 31673176 PMC6804388

[19] Eamsobhana P Chalayon O Kaewpornsawan K Ariyawatkul T. Missed Monteggia fracture dislocations treated by open reduction of the radial head. Bone Joint J. (2018) 100-B(8):1117–24. 10.1302/0301-620X.100B8.BJJ-2017-0866.R3 30062935

[20] Stragier B De Smet L Degreef I. Long-term follow-up of corrective ulnar osteotomy for missed Monteggia fractures in children. J Shoulder Elbow Surg. (2018) 27(11):e337–43. 10.1016/j.jse.2018.06.029 30224208

[21] Gallone G Trisolino G Stilli S Gennaro D L G. Complications during the treatment of missed Monteggia fractures with unilateral external fixation: a report on 20 patients in a 10-year period in a tertiary referral center. J Pediatr Orthop B. (2019) 28(3):256–66. 10.1097/BPB.0000000000000592 30789537

[22] Chen H-Y Wu K-W Dong Z-R Huang S-C Kuo KN Wang T-M. The treatment of chronic radial head dislocation in Monteggia fracture without annular ligament reconstruction. Int Orthop. (2018) 42(9):2165–72. 10.1007/s00264-018-3943-6 29713746

[23] Baydar M Öztürk K Orman O Akbulut D Keskinbıçkı MV Şencan A. Use of corrective ulnar osteotomy and radial head relocation into preserved annular ligament in the treatment of radiocapitellar instability secondary to pediatric chronic Monteggia fracture-dislocation. J Hand Surg Am. (2022) 47(5):481.e1–e9. 10.1016/j.jhsa.2021.05.025 34253391

[24] Liu Y Zhao H Xu H Shi W Li J Li Y. To angulate or not to angulate the Ulna during the progressive distraction period performed with a monolateral external fixator in paediatric patients with a chronic Monteggia fracture? Medicina (Kaunas). (2022) 58(11):1666. 10.3390/medicina58111666 36422205 PMC9697305

[25] Xu Z Li Y Wang Z Cai H. Open reduction combined with CORA-based osteotomy of the ulna in the treatment of missed Bado type I Monteggia injury: a retrospective study of 5 cases. Medicine (Baltimore). (2017) 96(47):e8609. 10.1097/MD.0000000000008609 29381932 PMC5708931

[26] Aslan L Gedik CC Birsel O Eren I Gönen E Demirhan M. Functional outcomes of pediatric true and equivalent Monteggia fractures - review of the literature. Ulus Travma Acil Cerrahi Derg. (2023) 29(6):724–32. 10.14744/tjtes.2022.52042 37278069 PMC10315934

[27] Take M Tomori Y Sawaizumi T Majima T Nanno M Takai S. Ulnar osteotomy and the Ilizarov mini-fixator for pediatric chronic Monteggia fracture-dislocations. Medicine (Baltimore). (2019) 98(1):e13978. 10.1097/MD.0000000000013978 30608438 PMC6344151

[28] Dai Z-Z Xu J Zhang Z-Q Li H Jin F-C. Risk factors for redislocation of chronic Monteggia fracture-dislocation in children after reconstruction surgery. Int Orthop. (2022) 46(10):2299–306. 10.1007/s00264-022-05473-3 35697865

[29] Yuan Z Xu HW Liu YZ Li YQ Li JC Canavese F. The use of external fixation for the management of acute and chronic Monteggia fractures in children. J Child Orthop. (2019) 13(6):551–9. 10.1302/1863-2548.13.190115 31908671 PMC6924126

[30] Su P Wang S Lai Y Zhang Q Zhang L. Screw analysis, modeling and experiment on the mechanics of tibia orthopedic with the Ilizarov external fixator. Micromachines (Basel). (2022) 13(6):932. 10.3390/mi13060932 35744545 PMC9230680

[31] Bor N Rubin G Rozen N Herzenberg JE. Chronic anterior Monteggia lesions in children: report of 4 cases treated with closed reduction by ulnar osteotomy and external fixation. J Pediatr Orthop. (2015) 35(1):7–10. 10.1097/BPO.0000000000000203 24787311

[32] Fukuda M Hidaka N. Association of radial head dislocation with fracture and acute plastic deformation of the ulna: unreported subtype of Monteggia fracture-dislocation in children. J Pediatr Orthop B. (2021) 30(2):190–5. 10.1097/BPB.0000000000000783 32694439

[33] Langenberg LC Beumer A The B Koenraadt K Eygendaal D. Surgical treatment of chronic anterior radial head dislocations in missed Monteggia lesions in children: a rationale for treatment and pearls and pitfalls of surgery. Shoulder Elbow. (2020) 12(6):422–31. 10.1177/1758573219839225 33281947 PMC7689610

[34] Park H Park KW Park KB Kim HW Eom NK Lee DH. Impact of open reduction on surgical strategies for missed Monteggia fracture in children. Yonsei Med J. (2017) 58(4):829–36. 10.3349/ymj.2017.58.4.829 28540998 PMC5447116

